# Computational Pipeline for Glomerular Segmentation and Association of the Quantified Regions with Prognosis of Kidney Function in IgA Nephropathy

**DOI:** 10.3390/diagnostics12122955

**Published:** 2022-11-25

**Authors:** Yoshimasa Kawazoe, Kiminori Shimamoto, Ryohei Yamaguchi, Issei Nakamura, Kota Yoneda, Emiko Shinohara, Yukako Shintani-Domoto, Tetsuo Ushiku, Tatsuo Tsukamoto, Kazuhiko Ohe

**Affiliations:** 1 Artificial Intelligence in Healthcare, Graduate School of Medicine, The University of Tokyo, 7-3-1, Hongo, Bunkyo-ku, Tokyo 113-0033, Japan; 2 Ohshima Memorial Kisen Hospital, 3-5-15, Misaki, Chiba 274-0812, Japan; 3 NTT DOCOMO, Inc., Sanno Park Tower, 2-11-1, Nagata-cho, Chiyoda-ku, Tokyo 100-6150, Japan; 4 Department of Reproductive, Developmental, and Aging Sciences, Graduate School of Medicine, The University of Tokyo, 7-3-1, Hongo, Bunkyo-ku, Tokyo 113-0033, Japan; 5 Department of Diagnostic Pathology, Nippon Medical School Hospital, 1-1-5, Sendagi, Bunkyo-ku, Tokyo 113-8602, Japan; 6 Department of Pathology, Graduate School of Medicine, The University of Tokyo, 7-3-1, Hongo, Bunkyo-ku, Tokyo 113-0033, Japan; 7 Department of Nephrology and Dialysis, Tazuke Kofukai Medical Research Institute, Kitano Hospital, 2-4-20, Ohgimachi, Kita-ku, Osaka 530-8480, Japan; 8 Department of Biomedical Informatics, Graduate School of Medicine, The University of Tokyo, 7-3-1, Hongo, Bunkyo-ku, Tokyo 113-0033, Japan

**Keywords:** computer vision, deep learning, digital pathology, whole slide imaging (WSI), object detection, segmentation, kidney disease, IgA nephropathy, glomerular sclerosis, renal prognosis

## Abstract

The histopathological findings of the glomeruli from whole slide images (WSIs) of a renal biopsy play an important role in diagnosing and grading kidney disease. This study aimed to develop an automated computational pipeline to detect glomeruli and to segment the histopathological regions inside of the glomerulus in a WSI. In order to assess the significance of this pipeline, we conducted a multivariate regression analysis to determine whether the quantified regions were associated with the prognosis of kidney function in 46 cases of immunoglobulin A nephropathy (IgAN). The developed pipelines showed a mean intersection over union (IoU) of 0.670 and 0.693 for five classes (i.e., background, Bowman’s space, glomerular tuft, crescentic, and sclerotic regions) against the WSI of its facility, and 0.678 and 0.609 against the WSI of the external facility. The multivariate analysis revealed that the predicted sclerotic regions, even those that were predicted by the external model, had a significant negative impact on the slope of the estimated glomerular filtration rate after biopsy. This is the first study to demonstrate that the quantified sclerotic regions that are predicted by an automated computational pipeline for the segmentation of the histopathological glomerular components on WSIs impact the prognosis of kidney function in patients with IgAN.

## 1. Introduction

The number of patients who are on dialysis due to end-stage renal failure is increasing worldwide, which has become a major health economic problem. According to a recent report [1], the number of patients undergoing chronic dialysis worldwide exceeded two million in 2010, and this number may double by 2030. The early detection and management of chronic kidney disease (CKD) is important in order to prevent its progression to end-stage renal failure. Immunoglobulin A nephropathy (IgAN) is the leading cause of CKD worldwide. It typically progresses to end-stage renal failure in 15–20% of patients after 10 years, and approximately 40% of patients after around 20 years [2,3]. Using evidence-based clinical practice guidelines in Japan [4], the clinical predictors for the progression of IgAN at the time of the initial renal biopsy include the following: (1) the presence of hypertension; (2) the amount of proteinuria with a usual cut-off of >1 g/day; (3) the degree of renal dysfunction; and (4) the histopathological grade, based on renal pathology. Of these predictors, histopathological findings play a key role but require observation by experts under a microscope. Patients with IgAN have varied histopathological lesions, ranging from mild mesangial proliferation, endocapillary hypercellularity, and crescentic glomerulonephritis to global and segmental sclerosis. For example, sclerosis represents the final appearance of glomerular injury that is caused by various diseases. When sclerosis occurs globally, determining the cause of the injury can be difficult.

Two histopathological grading systems are referred to in the clinical guidelines. The first system is the Oxford classification [5,6], which is based on the score of mesangial hypercellularity (M: M0, ≤0.5; M1, >0.5), endocapillary hypercellularity (E: E0, absent; E1, present), segmental sclerosis (S: S0, absent; S1, present), tubular atrophy or interstitial fibrosis (T: T0, 0–25%; T1, 26–50%; T2, >50%), and cellular or fibrocellular crescents (C: C0, absent; C1, 0–25%; C2, >25%). The second system is the Japanese histological grade classification (H-Grade) [7,8], which is based on the presence of acute lesions (i.e., cellular crescent, tuft necrosis, and fibrocellular crescent) and chronic lesions (i.e., global sclerosis, segmental sclerosis, and fibrous crescent). Detecting these complex findings among all of the glomeruli in whole slide images (WSIs) is laborious and time consuming, even for highly trained pathologists or nephrologists. Furthermore, the assessment is not always consistent [9,10]. Suppose the findings of all of the glomeruli on a WSI could be quantified with a computer, it may lead to a more thorough investigation of their impact on the prognosis of immunoglobulin A nephropathy (IgAN) and accelerate such research.

In the past decade, the number of studies aiming to develop deep learning applications for nephropathology has increased rapidly. Computational image recognition focusing on the glomerulus is generally classified into the following three types: the detection of glomeruli [11,12,13,14], the classification of the glomeruli [10,15], and the segmentation of the glomeruli [16,17,18,19,20,21,22,23,24]. The glomeruli that are detected in the WSI are localized by drawing bounding boxes. This approach would be a good application of automation because detecting glomeruli is simple but tedious for humans. Additionally, the development of such tools is realistic, as previously reported [13]. The classification of glomeruli, such as the presence or the absence of certain pathological findings, is more challenging because it requires the interpretation of quantitative histopathological lesions into qualitative expressions, for which expert assessment is not always consistent [9,10]. The segmentation of glomeruli localizes and quantifies every glomerulus by identifying the regions of each glomerulus in the pixels. Several studies have attempted to distinguish between the entire glomerulus and the background [16,17] or to distinguish between the normal and the sclerotic glomeruli [20,21,24]. Other studies have focused on the tubules, the blood vessels, and the interstitium, in addition to the glomerulus [19,23], or on the components inside of the glomerulus [18,22]. Segmenting the glomerulus and its components would be more helpful for a better understanding of kidney disease because it will be applied in the classification of pathological findings and to develop a prognostic model by utilizing quantified histopathological regions. Table 1 shows the previous studies for glomerular segmentation from WSI.

As the configuration of segmentation tasks varies from researcher to researcher, the high performance of a machine learning model does not necessarily indicate its usefulness for subsequent analyses. Previous studies [18,19,22] have assessed the usefulness of segmentation results in subsequent analyses, whereas other studies have only assessed the performance of machine learning models. In addition, these previous studies have only [19] evaluated the performance of machine learning models against external WSI, whereas the other studies have evaluated a single facility. Due to their high performance, deep neural networks (DNNs) tend to overfit to minute differences in the images that are used for training. Furthermore, the pathological specimens differ between facilities due to the differences in the preparation protocols. These factors have a non-negligible impact on the generalizability of studies dealing with WSI in DNNs. Therefore, in assessing the performance of the developed DNNs, an internal evaluation using only the WSIs of a single facility is not sufficient; external evaluations of the WSIs of different facilities are also important. Based on these two points, we propose an automated computational pipeline to detect the glomeruli from periodic acid-Schiff (PAS)-stained WSI and to segment the Bowman’s space, the glomerular tuft, and the histopathological components of crescentic and sclerotic regions. The pipelines were developed using the WSIs of two facilities independently, and the performances across the facilities were evaluated. In order to assess the significance of the quantified histopathological regions, we conducted a multivariate regression analysis to determine whether the proportion of the sclerotic regions was significantly associated with the prognosis of kidney function in patients with IgAN.

## 2. Materials and Methods

### 2.1. Data Collection

**Figure A1:**
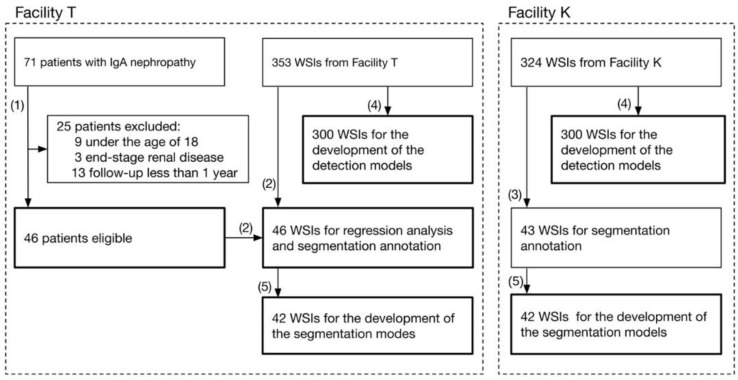
Overview of the data collection and selection procedure. (1) Eligibility criteria of immunoglobulin A nephropathy (IgAN) for regression analysis. (2) Facility T: whole slide image (WSI) selection for regression analysis and segmentation annotation. (3) Facility K: WSI selection for segmentation annotation. (4) WSI selection for the development of glomerular detection models. (5) WSI selection for the development of the glomerular segmentation models. Bold boxes indicate the data used in the experiments described below.

The Institutional Review Board approved all experiments and data collection at the University of Tokyo Hospital (Tokyo, Japan; approval number: 11455) and Tazuke Kofukai Medical Research Institute, Kitano Hospital (Osaka, Japan; approval number: P17-05-004). All of the experiments were conducted following the Ethical Guidelines for Medical and Biological Research Involving Human Subjects in Japan. Informed consent was obtained from all participants through opt-out on the website. (See Figure A1 in Appendix A for an overview of the data collection and selection).

#### 2.1.1. Collection of the WSIs from Two Facilities

The University of Tokyo Hospital (facility T) collected 353 PAS-stained WSIs of renal biopsy specimens from 2010 to 2016. From Kitano Hospital (facility K), 324 PAS-stained WSIs were collected from 2005 to 2017. In both facilities, various kidney diseases were included in the WSIs, and the slide digitization was conducted using a NanoZoomer C9600-12 slide scanner (Hamamatsu Photonics, Hamamatsu City, Shizuoka, Japan) with a 40× objective at a resolution of 0.23 μm/pixel.

#### 2.1.2. Eligible IgAN Cases for the Regression Analysis

For the regression analysis for the prognosis of kidney function in IgAN cases, the data of 71 patients with IgAN, who had undergone a renal biopsy between 2010 and 2016 at facility T, were collected from their electronic health records (EHRs), which included information on their age, sex, diagnosis, blood, and urine test findings, and clinical records. Among these patients, those who met the following criteria were excluded: (1) <18 years at the time of the biopsy, (2) end-stage renal failure (e.g., maintenance hemodialysis, kidney transplantation, or estimated glomerular filtration rate (eGFR) <15 mL/min/1.73 m^2^) at the time of biopsy, and (3) <1 year of eGFR follow-up after the biopsy. The data of 46 patients with IgAN were ultimately eligible for the regression analysis. Table 2 shows the statistical summary of the patients with IgAN.

### 2.2. Ground Truth Annotations

An assistant manually annotated the glomerular regions by bounding boxes in the 353 WSIs from facility T and the 324 WSIs from facility K using a computer-based commercial tool (RectLabel; available at https://rectlabel.com accessed on 19 November 2022) under the supervision of a nephrologist and a physician. The annotation for glomerular detection by bounding boxes requires the location of four vertex and class labels as supervised data. The average number of glomeruli in the WSI from facility T and facility K was 34 per WSI and 26 per WSI, respectively.

The annotation for segmentation requires assigning each pixel in an image to a specific class of object. We assigned each pixel of the cropped glomerular images to the following five classes: Bowman’s space, glomerular tuft, crescentic region, sclerotic region, and background. The inner region surrounded by Bowman’s capsule was annotated as a Bowman’s space, and the area containing the glomerulocapillaries and intraglomerular mesangium region was annotated as a glomerular tuft. The crescentic and sclerotic regions were annotated using our previously developed criteria [10]. According to these criteria, there are three types of crescents, namely the “fibrous crescent,” “fibrocellular crescent,” and “cellular crescent.” However, we did not distinguish between these crescentic regions in this study. “Sclerosis” comprises “capillary collapse,” “segmental sclerosis,” and “global sclerosis”; similarly, we did not distinguish between these sclerotic regions.

**Figure 1:**
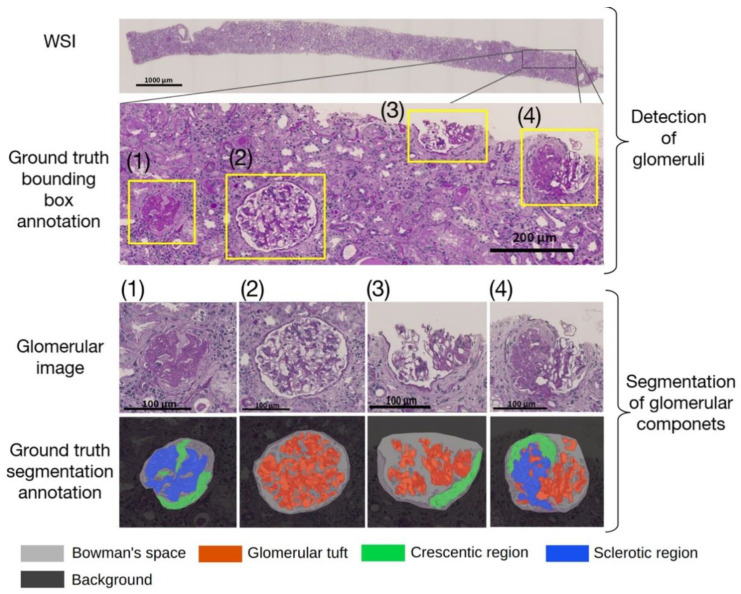
Example of a whole slide image (WSI) (**top row**). The bounding boxes of a glomerulus are shown as a rectangle with a yellow border in the second row. The glomerular images cropped by the bounding boxes are shown in the third row. The annotated images for the segmentation corresponding to the cropped glomeruli are shown in the bottom row. The examples in the (**bottom row**) (1)–(4) include cases with different percentages of sclerotic regions. (1) is an example of global sclerosis, in which there is no glomerular tuft (red). (2) and (3) are examples without sclerosis, in which there is no sclerotic region (blue). (4) is an example of segmental sclerosis, in which the glomerular tuft (red) and sclerotic region (blue) are almost equal in area.

A nephrologist and a pathologist depicted paper-based annotation drafts in the 46 WSIs from facility T and the 43 WSIs from facility K. Two assistants also performed the annotations using a computer-based tool (labelme; available at https://github.com/wkentaro/labelme accessed on 19 November 2022). Figure 1 illustrates examples of annotation for glomerular detection and segmentation. Table 3 shows the characteristics of the dataset that was used for glomerular segmentation.

### 2.3. Computational Pipeline

**Figure 2:**
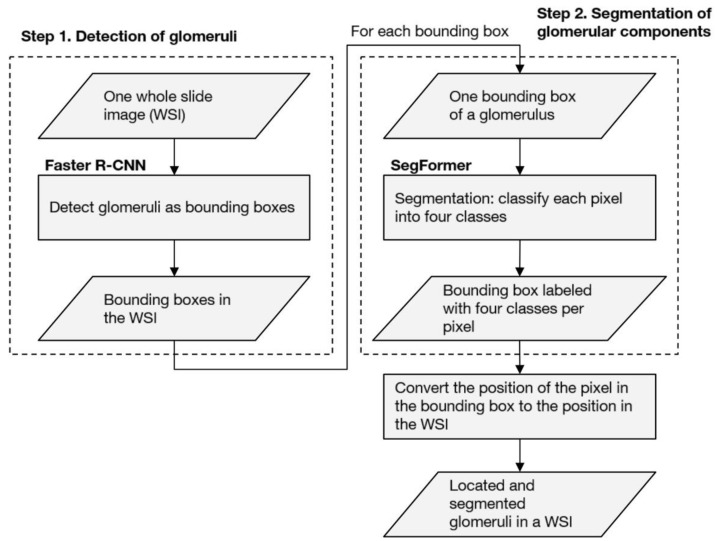
Overview of the computational pipeline. The parallelograms indicate the input or output data. The rectangles indicate the process. Faster R-CNN is described by Ren et al. [25], and SegFormer is described by Xie et al. [26].

To segment the histopathological regions inside the glomeruli from a high-resolution WSI, we developed a computational pipeline comprising the following two steps: (1) the detection of glomeruli, which draws bounding boxes surrounding the glomeruli in a WSI using Faster R-CNN, as described by Ren et al. [25], and (2) the segmentation of glomerular components, which classifies image pixels in bounding boxes into five classes (i.e., Bowman’s space, glomerular tuft, crescentic region, sclerotic region, and background) using SegFormer, as described by Xie et al. [26], which is a transformer-based [27] state-of-the-art segmentation method. All of the pixels that were detected as “not glomerulus” in the first step were assigned to the background. The labels of each pixel that were calculated in step 2 were repositioned in the WSI to compose the results of the entire WSI. Figure 2 shows an overview of the computational pipeline. 

#### 2.3.1. Step 1: Detection of Glomeruli

Faster R-CNN with a sliding window, as presented in [13], was applied. All of the WSIs were downsampled from 40× magnification to 5× magnification to balance the detection accuracy and processing speed. To train the model, images that were cropped by 2000 µm-square windows centered on each annotated glomerulus were used. Incomplete glomerular bounding boxes at the boundaries of the windows were ignored. Data augmentation techniques (e.g., flipping, Gaussian blurring, and sharpening) were applied to train the network to improve its robustness for variations in morphology and staining. The entire WSI was scanned with a sliding window (row-by-row, left-to-right) to evaluate the model. Each image of the sliding window was fed into the model. Neighboring windows overlapped each other by 10% (i.e., 200 µm), such that all of a glomerulus could be included in a window, even if it was at the boundary of the window. When a detected glomerulus was in the overlapping region of the neighboring windows, the bounding boxes that were overlapping by 35% or more were merged into one.

#### 2.3.2. Step 2: Segmentation of the Glomerular Components

SegFormer was used to segment the glomerular components, which classified each pixel of a glomerular image into the following five classes: Bowman’s space, glomerular tuft, crescentic region, sclerotic region, and background. To train the model, manually cropped glomerular images with an added margin of 20 μm were used to facilitate the easy training of the features outside of the glomerulus. This 20 μm margin width was set to 1/10 of the 200 μm, which is the estimated diameter of a glomerulus. When evaluating the model after glomerular detection, a 20 μm margin was added to the obtained image so that it would be similar to the training image. Data augmentation techniques (e.g., flipping, scaling, cropping, changing contrast, Gaussian blurring, and sharpening) were applied. The “Method details” section in Appendix G describes the critical aspects of Faster R-CNN and SegFormer and the evaluation metrics.

### 2.4. Multivariate Analysis for eGFR Prognosis in IgAN

The WSI and clinical information of 46 eligible patients with IgAN from facility T were analyzed (see Figure A1(1)). The prognostic variables were as follows: (1) age at biopsy, (2) sex, (3) presence or absence of prebiopsy hypertension, (4) eGFR at biopsy, (5) urine protein–creatinine ratio (UPCR) at biopsy, and (6) the mean proportion of the sclerotic regions compared to the whole glomerular regions in a WSI. For the histopathological variables, we used the proportion of the area of the sclerotic regions compared to the combined area of glomerular tuft and sclerotic regions.

The whole glomerular tuft region was obtained by combining the glomerular tuft and sclerotic regions. Variables 1–5 were obtained from the EHRs, and variable 6 was obtained with the developed computational pipeline by calculating the proportion of a sclerotic region to the whole glomerular tuft for all glomeruli in a WSI. The whole glomerular tuft was calculated as the sum of the glomerular tufts and sclerotic regions in the glomerular image. For the objective variable, we used the eGFR slope that was calculated from eGFRs within 2 years after renal biopsy. The eGFR slope was the slope of the univariate linear regression model of eGFR over time. This outcome represents a more dynamic tendency, compared to measurements taken at one point [28,29,30,31,32,33]. Multivariate regression analysis was conducted to assess the impact of the prognostic factors on the eGFR slope by estimating the partial regression coefficients and their *p*-values. Multicollinearity between the prognostic variables was assessed using VIF statistics.

### 2.5. Experiment Settings

To consider the mutual applicability between the facilities, computational pipelines were developed by independently using WSIs from facility T and facility K and evaluating the performances across the facilities. The details of the cross-validation settings and evaluation across facilities are described in the Appendix G.1.7. 

## 3. Results

### 3.1. Performance of the Computational Pipeline

#### 3.1.1. Glomerular Detection

**Figure A2:**
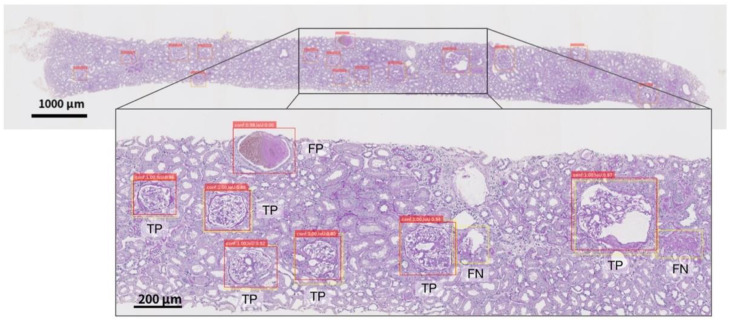
Example of glomerular detection results from a WSI. The yellow frames indicate the ground truth of the glomerular. The red frames indicate the predicted glomerular regions proposed by Faster R-CNN. The regions with overlapped yellow and red frames indicate a true positive (TP), the regions surrounded by only red frames indicate a false positive (FP), and the regions surrounded by only yellow frames indicate a false negative (FN).

As for the results of the glomerular detection alone, the F1 score (standard error) of the model that was trained with the WSIs of facility T (educational university hospital) for the WSIs of facility T (i.e., T to T) was 0.919 (0.003). The F1 score of the model that was trained with the WSIs of facility K (general hospital and research center) for the WSIs of facility K (i.e., K to K) was 0.912 (0.009). No significant difference existed between these F1 scores (*p* = 0.08), indicating no difference in the model’s performance against the WSI of its facility, which has been referred to as “internal performance.” In contrast, the F1 scores of T to K and K to T were 0.892 (0.005) and 0.875 (0.009), respectively. Significant differences existed between the scores of T to T and T to K (*p* < 0.01) and between K to K and K to T (*p* = 0.01). These results have revealed that, in both models, the performance decreased against the external facility’s WSI, which has been referred to as “external performance.” Table A1 in Appendix B shows the performance of glomeruli detection. Figure A2 in Appendix C depicts an example of the results of glomerular detection on a WSI.

#### 3.1.2. Glomerular Segmentation and the Pipeline

**Figure 3:**
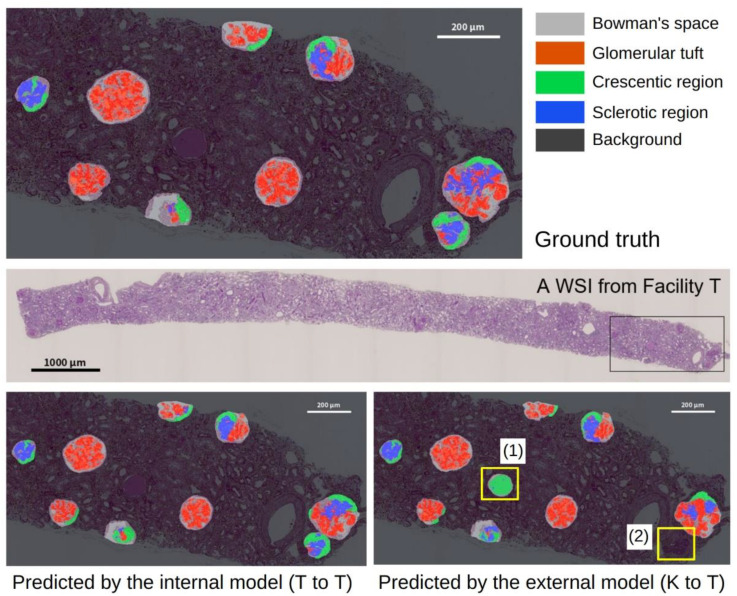
Example of the results of a whole slide image (WSI) from facility T. **Top**: manually annotated glomeruli and their components (i.e., ground truth). Middle: The WSI of the renal sample. The box is the area depicted in the top and bottom images. **Bottom left**: The predicted result obtained by model T (i.e., internal model). **Bottom right**: The predicted result obtained by model K (i.e., external model). In the example of the predicted result in the bottom right (K to T), (1) a dilated tubule filled with Tamm–Horsfall protein is incorrectly detected as glomerulus, and (2) a glomerulus is undetected, both of which are due to errors that occurred in the detection process in the first step of the computational pipeline.

**Figure 4:**
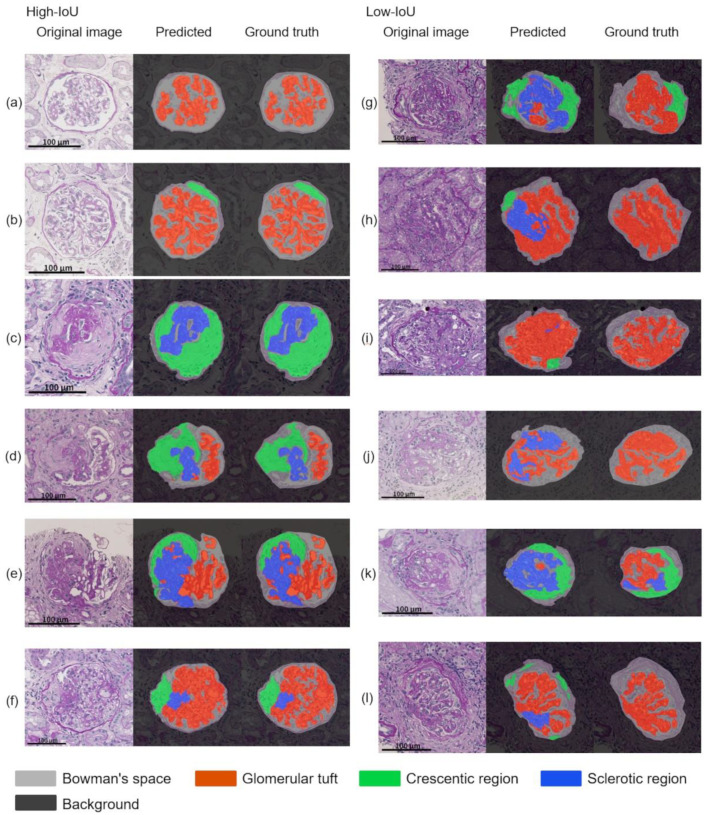
Example of the glomerular component segmentation obtained by the pipeline of K to T (predicted by the external model). The left column shows examples with higher mean intersection over union (IoU) of (**a**) 0.894, (**b**) 0.879, (**c**) 0.846, (**d**) 0.818, (**e**) 0.792, and (**f**) 0.780. The right column shows examples with lower mean IoU of (**g**) 0.377, (**h**) 0.384, (**i**) 0.412, (**j**) 0.417, (**k**) 0.438, and (**l**) 0.453. In the right column (**a**–**f**), the Bowman’s space, glomerular tuft, crescentic, and sclerotic region are correctly segmented. The pairs of the proportions of the sclerotic region of ground truth that are predicted in left column are (**a**–**c**) 0.0 to 0.0, (**d**) 0.485 to 0.446, (**e**) 0.451 to 0.518, and (**f**) 0.085 to 0.101, which are generally corresponding. (**g**) Most of the glomerular tuft region is incorrectly segmented as the sclerotic region, and the left side of the Bowman’s spaces are incorrectly segmented to the crescentic regions. (**h**) The left side of the glomerular tuft region is incorrectly segmented to the sclerotic region, and the upper left Bowman’s space is incorrectly segmented as the crescentic region. (**i**) The Bowman’s spaces in the glomerular tuft gaps are incorrectly segmented as the glomerular tuft areas, and the bottom of the glomerular tuft is incorrectly segmented as the crescentic regions. (**j**) The upper and lower left glomerular tuft areas are incorrectly segmented as the sclerotic regions. (**k**) Most of the glomerular tuft region from the upper left to the center is incorrectly segmented to the sclerotic region. (**l**) The lower left glomerular tuft region near the vascular pole is incorrectly segmented to the sclerotic region, and several small regions around the Bowman’s space are incorrectly segmented to the crescentic regions. The pairs of the proportions of the sclerotic region of ground truth that are predicted in right column are (**g**) 0.0 to 0.832, (**h**) 0.0 to 0.282, (**i**) 0.0 to 0.009, (**j**) 0.0 to 0.430, (**k**) 0.247 to 0.967, and (**l**) 0.0 to 0.158. All scale bars indicate 100 mm.

The top of Table 4 presents the segmentation performance. The mean (standard error (SE)) intersection over union (IoU) of T to T and K to K were 0.741 (0.011) and 0.764 (0.016), respectively. This finding indicated no significant difference between the internal performance of each model (*p* = 0.285). However, the external performance of the models tended to decrease. The mean IoU (SE) of K to T was 0.682 (0.002), which was lower than the mean IoU (SE) of T to T [0.741 (0.011)], and showed a significant difference (*p* = 0.003). The mean IoU of T to K was 0.737 (0.005), which was lower than the mean IoU of K to K [0.764 (0.016)], but the difference was not significant (*p* = 0.164). The bottom of Table 4 shows the segmentation performance after the detection (i.e., pipeline). The pipeline results were generally lower than those of the segmentation alone, owing to the accumulated error in the detection. As in the cases of segmentation alone, no significant difference existed in the mean IoU between T to T (0.670) and K to K (0.693), which indicated no difference in their internal performance (*p* = 0.395). In addition, the external performance of the models tended to decrease, as in the case of segmentation alone. The mean IoU of K to T was 0.609 (0.002), which was lower than the mean IoU of T to T [0.670 (0.017)], and showed a significant difference (*p* = 0.015). The mean IoU of T to K was 0.678 (0.002), which was lower than the mean IoU of K to K [0.693 (0.020)], but the difference was not significant (*p* = 0.509). Figure 3 depicts an example of the results that were obtained by the pipeline of T to T and K to T. Some examples of glomeruli with a high or low mean IoU that were obtained by the pipeline of K to T are shown in Figure 4.

#### 3.1.3. Regression Analysis for Kidney Prognosis

Table 5 shows the results of the multivariate analysis of the estimated glomerular filtration rate (eGFR) slope within two years after renal biopsy in 46 patients with IgAN. The column of the ground truth shows the results when manually annotated regions of the glomerular tuft and the sclerotic region were used. The columns of T to T and K to T show the results when each pipeline’s predicted sclerotic regions were used. The coefficients of determination (R^2^) for the ground truth, T to T, and K to T models were 0.18, 0.17, and 0.16, respectively. For multicollinearity, no variable had a variance inflation factor (VIF) value of > 3.0 in the ground truth model. In all of the models, the proportion of the sclerotic regions had a significant negative impact on the eGFR slope (*p* < 0.05). However, no other variables showed a significant impact. The results of the univariate regression analysis showed the same tendency (see Table A2 in Appendix D).

**Figure A3:**
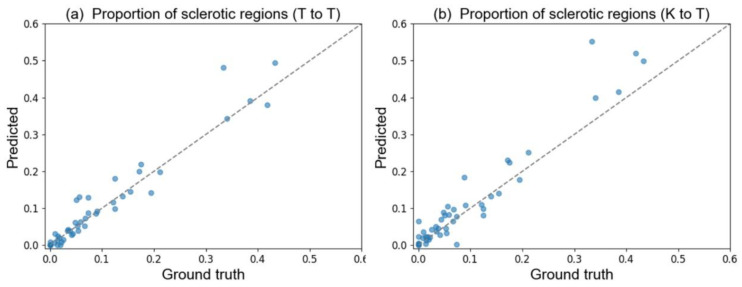
Scatter plots of the proportion of sclerotic regions between the ground truth and predicted regions. (**a**) Sclerotic regions of T to T. (**b**) Sclerotic regions predicted by K to T. “T to T” is the result of the facility T model for the WSIs of facility T. “K to T” is the result of the facility K model for the WSIs of facility T.

Table A3 in Appendix E presents the correlation coefficients between the ground truth regions and the predicted regions by the pipeline for the sclerotic and the semicircular regions in 46 IgAN cases. The results were high values that exceeded 0.96. The scatter plots for the sclerotic regions in the T to T and the K to T models are shown in Figure A3 in Appendix F.

## 4. Discussion

In this paper, we describe an automated computational pipeline that can detect glomeruli in PAS-stained WSI and segment the histopathological components inside of the glomerulus. Based on multivariate analysis, the predicted sclerotic regions, even the regions that were predicted by the external model, had a significant negative impact on the eGFR slope within two years after biopsy. We believe that this study is the first to demonstrate the usefulness of an automated computational pipeline for segmenting the histopathological glomerular components on WSIs and demonstrate that quantified sclerotic regions impact the prognosis of the kidney function in patients with IgAN.

Several studies [18,19,20,21] aiming for pixel-level semantic segmentation for WSI of renal tissue sections have set the task of distinguishing between nonsclerotic and sclerotic glomeruli. Bueno et al. [20] sequentially applied SegNet-VGG19 [34] in order to segment glomeruli and applied AlexNet to classify them as nonsclerotic or sclerotic glomeruli. The segmentation accuracies for the nonsclerotic and the sclerotic were 96.06% and 83.22%, respectively. Hermsen et al. [19] evaluated U-Net-based 11 class segmentation, as described by Ronneberger et al. [35]. The normal glomeruli, sclerotic glomeruli, empty Bowman’s capsules, tubules, arteries, interstitium, and the capsules were fully annotated. The Dice coefficients of the normal and the sclerotic glomeruli were 0.95 and 0.62, respectively. Altini et al. [21] conducted SegNet-based semantic segmentation of nonsclerotic and sclerotic glomeruli; their IoUs were 0.66546 and 0.49215, respectively. Jiang et al. [24] conducted a mask region-based convolutional neural network (R-CNN)-based semantic segmentation for classifying glomeruli with a normal structure, an abnormal structure, and global sclerosis; the mean IoU for PAS-stained WSIs were 0.697, 0.544, and 0.646, respectively. The results of these previous studies could help us to quantify global glomerulosclerosis, the ratio between sclerotic glomeruli, and the overall number of glomeruli. However, because glomerular sclerosis does not always occur globally, pixel-level segmentation for partially sclerosed regions is required for detailed quantification. Such quantification should have an essential role in understanding kidney diseases.

As shown in Table 4, the performance of the segmentation alone and the pipeline showed no significant differences in the mean IoU between T to T and K to K. This finding indicated that their internal performances were comparable. This finding supports that the annotation for glomerular detection and segmentation was conducted with a constant quality. Compared to the performance of the models that were trained with internal WSIs, the performance of the models that were trained with external WSIs tended to decrease in the segmentation alone and the pipeline. One of the reasons for this finding may be due to differences in the slide preparation to the digitization process between the facilities. The differences in the staining protocols, the manufacturing processes, and the digital scanner processing between the laboratories caused minute differences in the WSIs; however, the pathological samples were stained similarly. This difference is imperceptible to the human eye, but it is sufficient to affect deep learning-based applications [36,37,38]. We applied color normalization in the preprocessing step and Gaussian blurring, sharpening, and contrast changes during the data augmentation. However, extended methods are required in order to compensate for the minute differences in WSIs between the facilities, which increases the robustness against external WSI. The successful adaptation of WSI in deep neural network-based applications depends on each step of high-quality pathology slide preparation, such as embedding, cutting, staining, and scanning [39,40], as well as color variations. Using precise and homogeneous WSIs is desirable; however, such a model may not necessarily be robust against external WSIs that have more diversity. Improving the interfacility applicability of the developed model is an important issue for the success of deep learning applications in digital pathology. In addition, the performance of K to T is significantly lower for both the segmentation alone and the pipeline, while the performance degradations of T to K are not significant. This may be because a small number of glomerular images (1011) were used to develop the segmentation in model K, compared to the number of glomerular images that were used to develop model T (1713). We used the same number of WSIs from both of the facilities for the segmentation task. However, the number of images differed because of the different number of glomeruli that were contained in each WSI. The relatively small number of glomerular images in the training data for model K may have resulted in less diversity, leading to the significant performance degradation of K to T.

As shown in Table 5, the manually quantified (ground truth) sclerotic regions were associated with negatively impacting the eGFR slope in the multivariate analysis. Segmental sclerosis, which is defined by the Oxford Classification [5,6], or the chronic lesions including segmental sclerosis and global sclerosis, which are defined by the H-Grade [7,8] have a negative impact on the poor prognosis of IgAN; however, the current study showed that the quantified sclerotic regions also have a negative impact on the eGFR slope within two years after biopsy. In our analysis, the effect of the post-biopsy treatment on eGFR was not adjusted because of the retrospective design, which is a limitation of this analysis. In addition, other limitations of this analysis were that the 2-year period was relatively short and the number of IgAN cases (*n* = 46) was also limited; these may have affected the relatively low coefficients of determination (0.18 in the ground truth model).

Table 5 also shows the same tendency in the standardized partial regression coefficients among the ground truth, the T to T (i.e., internal model), and the K to T (i.e., external model) models. The correlation between the ground truth regions and the predicted regions in each WSI aids in the understanding of their impact in the regression model. In Table A3 in Appendix E, the correlation coefficient for the sclerotic regions exceeded 0.96, even when using the external model. This finding indicated that the estimation of the total amount of sclerotic and glomerular tuft regions in each WSI was approximately correct. In light of the previous results, our developed pipeline shows a certain level of robustness for quantifying the glomerular tuft and sclerotic regions from WSI, even if the model is applied to the WSI of external facilities.

Another limitation of this study is that the concordance of the ground truth labels that have been used for developing glomerular detection and segmentation was not evaluated; however, the experts provided them. Surrounding the glomeruli with bounding boxes and drawing their histopathological components required distinguishing unclear boundaries with an understanding of pathology. Such labeling could vary among experts. Well-annotated examples are important in supervised learning; the main challenge in deep neural network-based applications for digital histopathology is obtaining high-quality labels. We carefully conducted the annotation with multiple experts, including a nephrologist and a pathologist, however the possibility of errors does exist. Nonetheless, annotation errors are not specific to this research; however, they should be kept in mind in studies on supervised learning.

## 5. Conclusions

We developed an automated computational pipeline for detecting glomeruli on PAS-stained WSIs, followed by segmenting the Bowman’s space, the glomerular tuft, the crescentic, and the sclerotic region inside of the glomeruli. The internal and external evaluation of the pipeline using WSIs from two facilities showed that the mean IoU of five regions, including the background, was 0.670 (T to T) and 0.693 (K to K) in the internal evaluation, and 0.609 (K to T) and 0.678 (T to K) in the external evaluation. The multivariate analysis for eGFR prognosis in cases of IgAN showed that the proportion of sclerotic regions that were quantified by the pipelines, even those that were quantified by the external model, had a significant negative impact on the eGFR slope, while five other clinical prognostic factors (i.e., age, sex, hypertension, eGFR at biopsy, and UPCR at biopsy) had no significant impact. These findings suggest the importance of quantifying the sclerotic region, as well as the usefulness and the robustness of the developed pipeline, for the purpose of predicting eGFR in cases of IgAN. The developed pipeline could aid in diagnosing renal pathology by visualizing and quantifying the histopathological feature of glomerulus. In addition, this high-throughput approach could potentially accelerate research in order to better understand the prognosis of IgAN.

## Figures and Tables

**Table 1:** Previous studies for glomerular segmentation from WSI.

Author	Year	Object	Method	Subsequent Analysis	Extrapolation Evaluation
Kato et al. [16]	2015	Glomerulus	S-HOG + SVM	-	-
Gallego et al. [17]	2018	Glomerulus	CNN	-	-
Ginley et al. [18]	2019	Glomerulus and Internal components ((1) a nuclear component; (2) a PAS-positive component consisting of mesangium, glomerular basement membranes, and Bowman’s capsule; (3) a luminal component consisting of Bowman’s space and capillary lumina)	Deep Lab v2	Tervaert classification and classification scheme defined by authors	-
Hermsen et al. [19]	2019	Renal structures (glomerulus, sclerotic glomerulus, empty Bowman’s capsules, proximal tubule, distal tubule, atrophic tubule, undefined tubule, artery, interstitium, and capsule)	U-Net	Banff classification	Radboud University and Mayo Clinic
Bueno et al. [20]	2020	Glomerulus (normal, sclerosed)	SegNet and U-Net	-	-
Antini et al. [21]	2020	Glomerulus (normal, sclerosed)	SegNet and Deeplab v3+	-	-
Zeng et al. [22]	2020	Glomerulus (global sclerosis, segmental sclerosis, crescent, or none of the above) and intraglomerular structures (mesangial cells, endothelial cells, and podocytes)	U-Net, DenseNet, LSTM-GCNet, and 2D V-Net	Mesangial hypercellularity score	-
Bouteldja et al. [23]	2021	Renal structures (glomerular tuft, glomerulus including Bowman’s capsule, tubules, arteries, arterial lumina, and veins)	U-Net	-	-
Jiang et al. [24]	2021	Glomerulus (normal, global sclerosis, and glomerular with other lesions)	Mask R-CNN	-	-

**Table 2:** Statistical summary of the patients with IgAN.

Facility	Case	Age (Median [IQR])	Sex (Female:Male)	Hypertension (Absent:Present)	eGFR (Median [IQR])	UPCR (Median [IQR])
T	46	42, [32, 61]	21:25	18:28	65.15 [45.80, 83.88]	1.18 [0.64, 2.39]

IgAN, immunoglobulin A nephropathy; IQR, interquartile range; eGFR, estimated glomerular filtration ratio (mL/min/1.73 m^2^); UPCR, urine protein–creatinine ratio (*g*/*g*).

**Table 3:** Characteristics of the annotated WSI for glomerular segmentation.

Facility	WSI	Number of Glomeruli (Total; Median [IQR])	Percentage of Crescentic Regions to Glomerulus (Median [IQR])	Percentage of Sclerotic Regions to Glomerulus (Median [IQR])
T	46	1713; 27.5 [20, 39.5]	3.46 [1.41, 6.47]	2.57 [0.74, 6.49]
K	42	1011; 24.0 [15, 30]	4.78 [1.39, 10.27]	5.37 [1.36, 9.06]

WSI, whole slide image; IQR, interquartile range.

**Table 4:** Performance of glomerular segmentation.

Evaluation Scope	Model to WSI	Background	Bowman’s Space	Glomerular Tuft	Crescentic Region	Sclerotic Region	Mean IoU
Segmentation Alone	T to T	0.965	0.664	0.770	0.596	0.707	0.741
		(0.001)	(0.009)	(0.006)	(0.032)	(0.021)	(0.011)
	K to K	0.973	0.696	0.810	0.665	0.674	0.764
		(0.002)	(0.013)	(0.014)	(0.033)	(0.032)	(0.016)
	*p*	0.028 *	0.094	0.037 *	0.160	0.418	0.285
	T to T	0.965	0.666	0.770	0.596	0.707	0.741
		(0.001)	(0.009)	(0.006)	(0.032)	(0.021)	(0.011)
	K to T	0.956	0.586	0.738	0.523	0.604	0.682
		(0.000)	(0.016)	(0.002)	(0.047)	(0.006)	(0.002)
	*p*	0.003 *	<0.001 *	0.004 *	0.070	0.004 *	0.003 *
	K to K	0.973	0.696	0.810	0.665	0.674	0.764
		(0.002)	(0.013)	(0.014)	(0.033)	(0.032)	(0.016)
	T to K	0.963	0.667	0.789	0.629	0.638	0.737
		(0.001)	(0.004)	(0.002)	(0.005)	(0.007)	(0.005)
	*p*	0.008 *	0.077	0.195	0.326	0.312	0.164
Segmentation after detection (i.e., pipeline)	T to T	0.999	0.594	0.736	0.497	0.521	0.670
		(0.000)	(0.014)	(0.015)	(0.041)	(0.023)	(0.017)
	K to K	0.999	0.626	0.768	0.530	0.540	0.693
		(0.000)	(0.020)	(0.017)	(0.039)	(0.038)	(0.020)
	*p*	NA	0.230	0.190	0.568	0.691	0.395
	T to T	0.999	0.594	0.736	0.497	0.521	0.670
		(0.000)	(0.014)	(0.015)	(0.041)	(0.023)	(0.017)
	K to T	0.999	0.509	0.683	0.412	0.442	0.609
		(0.000)	(0.005)	(0.001)	(0.003)	(0.003)	(0.002)
	*p*	NA	0.001 *	0.015 *	0.094	0.019 *	0.015 *
	K to K	0.999	0.626	0.768	0.530	0.540	0.693
		(0.000)	(0.020)	(0.017)	(0.039)	(0.038)	(0.020)
	T to K	0.999	0.602	0.749	0.516	0.527	0.678
		(0.000)	(0.002)	(0.002)	(0.006)	(0.003)	(0.002)
	*p*	NA	0.293	0.323	0.727	0.748	0.509

Unless otherwise specified, the data are presented as the mean (standard error). “T to T” represents the results from the facility T model against the facility T data. “K to K” represents the results from the facility K model against the facility K data. “T to K” is the result of the facility T model against facility K data. “K to T” is the result of the facility K model against the facility T data. * indicates a statistically significant difference (i.e., *p* < 0.05). The *p* value is based on Welch’s *t*-test for the equality of the means of two samples. WSI, whole slide image; NA not available; SE standard error.

**Table 5:** Results of the multivariate regression analysis.

	Ground Truth (R^2^ = 0.18)			T to T (R^2^ = 0.17)			K to T (R^2^ = 0.16)		
	Beta	*p* Value	VIF	Beta	*p* Value	VIF	Beta	*p* Value	VIF
Age	−1.289 (1.369)	0.34	1.9	−1.217 (1.376)	0.37	1.9	−1.242 (1.393)	0.36	1.9
Sex (male = 1)	−0.604 (1.144)	0.59	1.4	−0.550 (1.149)	0.62	1.3	−0.359 (1.151)	0.75	1.3
Hypertension (presence = 1)	−0.926 (1.194)	0.43	1.5	−0.999 (1.199)	0.39	1.5	−1.115 (1.211)	0.35	1.5
eGFR at renal biopsy (mL/min/1.73 m^2^)	−2.793 (1.554)	0.071	2.5	−2.727 (1.562)	0.079	2.5	−2.370 (1.540)	0.12	2.4
UPCR at renal biopsy (*g*/*g*)	0.211 (1.169)	0.85	1.4	0.024 (1.169)	0.98	1.4	0.069 (1.185)	0.95	1.4
Proportion of sclerotic regions (%)	−2.885 (1.207)	0.018 *	1.5	−2.732 (1.196)	0.024 *	1.5	−2.333 (1.126)	0.039 *	1.3

The data are presented as the mean (standard error), unless otherwise specified. “T to T” is the result of the facility T model applied to facility T data. “K to T” is the result of the facility K model applied to facility T data. * indicates a statistically significant difference (i.e., *p* < 0.05). The *p* value is based on Welch’s *t*-test for the equality of the means of two samples. Beta, standardized partial regression coefficient; R^2^, coefficient of determination; eGFR, estimated glomerular filtration rate; UPCR, urine protein–creatinine ratio; VIF, variance inflation factor.

## Data Availability

The datasets of the WSIs are unavailable to the public, and their use is restricted. The source code, network configurations, and trained network-derived results are available at the following URL: https://github.com/jinseikenai/glomeruli_segmentation accessed on 19 November 2022.

## Appendix B

**Table A1:** Performance of glomerular detection.

	T to T	K to K	*p*	T to T	T to K	*p*	K to K	K to T	*p*
F1 score	0.919 (0.003)	0.912 (0.009)	0.08	0.919 (0.003)	0.892 (0.005)	<0.01 *	0.912 (0.002)	0.875 (0.009)	0.01 *

F1 is the harmonic mean of precision and recall. “T to T” is the result of the facility T model against the WSIs of facility T. “K to K” is the result of the facility K model against the WSIs of facility K. “T to K” is the result of the facility T model against the WSIs of facility K. “K to T” is the result of the facility K model against the WSIs of facility T. * indicates a statistically significant difference (i.e., *p* < 0.05). The *p*-value is based on Welch’s *t*-test for equality of means of two results. Note: The data are presented as the mean F1 score (standard error).

## Appendix D

**Table A2:** Results of the univariate regression analysis.

	Ground Truth		T to T		K to T	
	Beta	*p*-Value	Beta	*p*-Value	Beta	*p*-Value
Age	−0.652 (1.014)	0.51	−0.652 (1.014)	0.51	−0.652 (1.014)	0.51
Sex (male = 1)	−0.368 (1.017)	0.71	−0.368 (1.017)	0.71	−0.368 (1.017)	0.71
Hypertension (present = 1)	−0.864 (1.010)	0.38	−0.864 (1.010)	0.38	−0.864 (1.010)	0.38
eGFR at renal biopsy (mL/min/1.73 m^2^)	−0.167 (1.018)	0.87	−0.167 (1.018)	0.87	−0.167 (1.018)	0.87
UPCR (*g*/*g*)	−0.479 (1.016)	0.63	−0.479 (1.016)	0.63	−0.479 (1.016)	0.63
Proportion of sclerotic regions (%)	−1.865 (0.975)	0.055	−1.786 (0.979)	0.067	−1.764 (0.980)	0.071

Unless otherwise specified, the data are presented as mean (standard error). “T to T” is the result of the facility T model applied to facility T data. “K to T” is the result of the facility K model applied to facility T data. The *p*-value is based on Welch’s *t*-test for the equality of the means of two samples. Beta, standardized partial regression coefficient; GFR, estimated glomerular filtration rate; UPCR, urine protein–creatinine ratio.

## Appendix E

**Table A3:** Correlation coefficients between the ground truth and predicted regions.

Model to WSI	Proportion of the Sclerotic Regions to the Combined Area of Glomerular Tuft and Sclerotic Regions
T to T	0.967
K to T	0.963

“T to T” is the result of the facility T model applied to facility T data. “K to T” is the result of the facility K model applied to facility T data. The correlation coefficient between T and T was derived from one of the six cross-validations. The correlation coefficient of “K to T” is the average of the six cross-validations.

## Appendix G

### Appendix G.1. Method Details

#### Appendix G.1.1. Faster R-CNN

A faster region-based convolutional neural network (Faster R-CNN [31]) is an object detection method that is based on convolutional neural networks (CNNs). Faster R-CNN consists of the following two modules: the region proposal network (RPN), which identifies the region of an object in an image, and a network that classifies the objects in the proposed region. Faster R-CNN first processes the input image by performing convolution and pooling layers in order to obtain feature maps and passes them to the RPN. The RPN then scans over the feature maps using a sliding window with different scales and aspect ratios and calculates two scores indicating whether each window contains an object and whether the object is a background or not. In order to solve the redundancy of the candidate regions that are obtained by the RPN, non-maximum suppression is used. The candidate regions with different sizes are converted into fixed-sized vectors through region of interest (ROI) pooling to be input into a fully connected layer. Finally, the coordinates and class information of the predicted multiple objects are output by performing fully connected layers.

#### Appendix G.1.2. SegFormer

SegFormer [34] is a semantic segmentation method that is based on transformers. Multiple semantic segmentation methods have been proposed. Most of them are based on CNNs, but recently, those that are based on transformers, which have been used in language models, have shown higher accuracy and are being used. SegFormer is an efficient and accurate semantic segmentation architecture among these transformer-based methods. It follows the encoder–decoder architecture. SegFormer consists of a hierarchical transformer encoder to extract coarse and fine features, and a lightweight all multi-layer perceptron (MLP) decoder. The performance of SegFormer may be slightly lower than some of the methods that require larger memory, such as Vision Transformer (ViT). However, SegFormer is significantly faster, with fewer model parameters than the other transformer-based architectures, and this feature is important for medical institutions without rich GPU resources.

#### Appendix G.1.3. Color Normalizations

In glomerular detection and segmentation, each RGB channel in a WSI was normalized by dividing the difference between the value of a pixel and the mean value of a pixel by the variance of the pixels. The mean and the variance were calculated from the training and validation datasets in order to train the network. The mean and the variance were calculated from the test dataset in order to test the network.

#### Appendix G.1.4. Evaluation Metrics

In order to evaluate the accuracy of glomerular detection, we calculated the micro average F1 score over all of the WSIs for each cross-validation trial. We used the average of these six cross-validations. If the IoU of the detected glomerulus and the ground truth were greater than 0.5, we classified the detection as a true positive (TP), and if the IoU was less than 0.5, we classified the detection as a false positive (FP). If the ground truth glomerulus had no overlapped predicted glomerulus with IoU ≥ 0.5, we classified the ground truth glomerulus as a false negative (FN). The equations that were used for the evaluation metrics were as follows (Equations (A1)–(A3)):

(A1) $$\mathrm{Recall}\;=\frac{\mathrm{TP}}{\mathrm{TP}\;+\;\mathrm{FN}}$$

(A2) $$\mathrm{Precision}\;=\frac{\mathrm{TP}}{\mathrm{TP}\;+\;\mathrm{FP}}$$

(A3) $$F1\;\mathrm{score}\;=\frac{2\times \mathrm{Recall}\;\times \mathrm{Precision}}{\mathrm{Recall}\;+\;\mathrm{Precision}}\;$$

In order to evaluate the accuracy of the glomerular component segmentation, we calculated the micro average IoU of each class over all of the WSIs for each trial, and we used the average of these six cross-validations. In this instance, the pixels in which the estimated label and the correct label matched are denoted as TP, the pixels with only the estimated class are denoted as FP, and the pixels with only the correct label are denoted as FN. The mean IoU was determined as the macro average of the IoU for each class (Equation (A4)) as follows:

(A4) $$\mathrm{IoU}\;=\frac{\mathrm{TP}}{\mathrm{TP}\;+\;\mathrm{FP}\;+\;\mathrm{FN}}$$

In evaluating the accuracy of the entire computational pipeline, we repositioned the predicted and correct segmentation labels on the entire WSI. We counted the number of TP, FP, and FN pixels over all of the WSIs that were included in the evaluation data in order to calculate the IoU (Equation (A4)). We evaluated the accuracy of the entire pipeline process using the averages of the six cross-validations.

#### Appendix G.1.5. Tools for Implementation

In order to develop the pipeline, we used Python, version 3.6.7 (Python Software Foundation, Wilmington, DE, USA), and the machine learning framework PyTorch version 1.7.1 (Facebook’s AI Research Lab, Menlo Park, CA, USA). We also implemented a Faster R-CNN that was provided by the PyTorch project and the implementation of SegFormer was provided by Hugging Face. The statistical analysis was conducted in R version 4.1.1 (R Foundation, Vienna, Austria), using the packages stats 4.1.1, tidyverse 1.3.1, ppcor 1.1, and car 3.0.11 cross-validation settings.

We split the entire dataset into training, validation, and test sets. The validation set was used for the model selection in order to avoid excessively fitting the model to the test set, which would impair the generalization performance. The test set was used in order to evaluate the performance. For glomerular detection, 300 WSIs in each facility were divided into six subsets by stratified splitting based on the number of glomeruli in each WSI. Six-fold cross-validations were conducted using 200 WSIs for the training, 50 WSIs for the validation, and 50 were used for the test.

Glomerular detection is a binary classification task that is evaluated by whether Faster R-CNN can correctly detect the bounding boxes surrounding the glomeruli given as ground truth. The micro average of the F1 score, which is the harmonic mean of precision and recall, was employed as an evaluation metric. In order to maximize the F1 score in the validation set, the threshold to distinguish between the glomerulus and the background was set. For the glomerular segmentation, 42 WSIs from each facility were divided into six subsets via stratified splitting based on the total pixels of the crescentic and sclerotic regions. Six-fold cross-validations were conducted using 28 WSIs for the training, 7 WSIs for the validation, and 7 WSIs for the test. Glomerular segmentation is a multiclassification task that is evaluated by whether SegFormer can correctly classify the pixels in an image into the ground truth labels. Micro averages of intersection over union (IoU) were employed as the evaluation metrics.

#### Appendix G.1.6. Hyperparameters

In Faster R-CNN, the hyperparameters are the same as they were in previous studies by the authors [13]. The optimizer that dynamically changed the learning rate used Momentum SGD; the learning rate was 0.0003, the momentum was 0.9, and the learning rate was reduced to 0.00003 after 900,000 iterations. Data augmentation techniques were also applied in order to train the network, which used a combination of vertical and horizontal flip. The training iterations were terminated by monitoring the F-measure of the validation set when the network had been trained sufficiently.

In SegFormer, the hyperparameters inherit those of the original SegFormer; the model size of SegFormer was mit-b4 [26], which has the second largest number of parameters due to limited computing resources and computational efficiency. An implementation by Hugging Face [41] was used. The batch size was set to 20 when training the model, and the model was selected at the epoch with the best mean IoU for the validation data, with an upper limit of 1000 epoch iterations.

#### Appendix G.1.7. Evaluation across Facilities

For glomerular detection and segmentation, six models that were developed using six-fold cross-validations were applied to all of the 300 or 42 WSIs of the other facilities. The average of each of the six times was assessed. For the evaluation of the computational pipelines, six pipelines consisting of six segmentation models, followed by one detection model, were applied to all 42 of the WSIs of the other facilities, and the average of the six times was assessed.
